# Dual Adaptive Filtering by Optimal Projection Applied to Filter Muscle Artifacts on EEG and Comparative Study

**DOI:** 10.1155/2014/374679

**Published:** 2014-09-14

**Authors:** Samuel Boudet, Laurent Peyrodie, William Szurhaj, Nicolas Bolo, Antonio Pinti, Philippe Gallois

**Affiliations:** ^1^Faculté de Médecine et Maïeutique, University Catholic of Lille, 59000 Lille, France; ^2^Unité de Traitement de Signaux Biomédicaux, 59000 Lille, France; ^3^Hautes Etudes d'Ingénieur, 13 Rue de Toul, 59000 Lille, France; ^4^Clinical Neurophysiology service of CHR of Lille, 59000 Lille, France; ^5^Harvard Medical School, Boston, MA 02115, USA; ^6^I3MTO-EA 4708, Université d'Orléans, 45000 Orléans, France; ^7^Groupe Hospitalier de l'Institut Catholique Lillois, 59000 Lille, France

## Abstract

Muscle artifacts constitute one of the major problems in electroencephalogram (EEG) examinations, particularly for the diagnosis of epilepsy, where pathological rhythms occur within the same frequency bands as those of artifacts. This paper proposes to use the method dual adaptive filtering by optimal projection (DAFOP) to automatically remove artifacts while preserving true cerebral signals. DAFOP is a two-step method. The first step consists in applying the common spatial pattern (CSP) method to two frequency windows to identify the slowest components which will be considered as cerebral sources. The two frequency windows are defined by optimizing convolutional filters. The second step consists in using a regression method to reconstruct the signal independently within various frequency windows. This method was evaluated by two neurologists on a selection of 114 pages with muscle artifacts, from 20 clinical recordings of awake and sleeping adults, subject to pathological signals and epileptic seizures. A blind comparison was then conducted with the canonical correlation analysis (CCA) method and conventional low-pass filtering at 30 Hz. The filtering rate was 84.3% for muscle artifacts with a 6.4% reduction of cerebral signals even for the fastest waves. DAFOP was found to be significantly more efficient than CCA and 30 Hz filters. The DAFOP method is fast and automatic and can be easily used in clinical EEG recordings.

## 1. Introduction

Electroencephalograms (EEG) remain essential in neurological practice; their indications are even increasing, especially for long-term EEG. EEG are captured continuously, sometimes during several days for hospitalized patient or for outpatients, in order to record paroxysmal clinical manifestations. EEG interpretation is difficult due to the low signal quality, specifically due to the numerous muscle artifacts interfering with the paroxysmal abnormalities detection or with the seizure analysis. Filters distributed with commercially available devices are insufficient. Either they do not eliminate enough muscle signal or they alter dramatically the cerebral signal. New automated filters are then required to better eliminate muscle artifacts, without altering cerebral signals.

**Figure 1:**
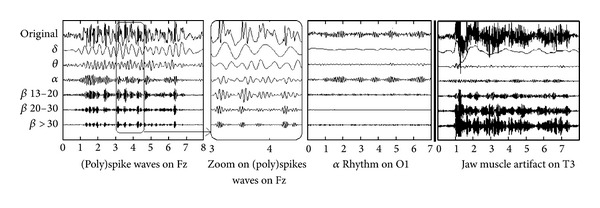
Frequency decomposition of cerebral rhythms and muscle artifacts [1].

Artifacts can have other origins including power source, eye movement/blinking, electrode, galvanic sudation, chewing, and heartbeat. This paper focuses on muscular contractions, which are the most important sources of artifacts under certain recording conditions. Muscle artifacts correspond to the electromyographic (EMG) potentials generated mainly by jaw and forehead muscles. For this reason, they are generally more important on the temporal and frontal channels. The major part of the signal power occurs at high frequencies(>13 Hz) (Figure 1).

The challenge for neurologists is to analyze brain signals masked by the artifacts in order to diagnose underlying pathologies. Brain signals measured on the scalp surface can be classified into four main frequency bands: Δ (0–4 Hz), *θ* (4–8 Hz), *α* (8–13 Hz), and *β* (13–30 Hz). Cortex may also generate gamma rhythms (>30 Hz), but these oscillations are of very low amplitude and are not classically observed in scalp EEG. In healthy awake adults, the EEG signal belongs mainly to the *α* band. In epileptic patients, particular rhythms can be observed including spikes (fast waves belonging to the *β* band, Figure 1), slow waves (0–8 Hz), or spike waves (a spike (>13 Hz) followed by a slow wave (<4 Hz)) (Figure 1). For a sleeping adult, other wave forms are observed (delta rhythms, K-complex, spindles, and vertex spikes). Muscular activity is also present during sleep, but EMG artifacts are rarer and rarely hamper the interpretation of EEG. It is still useful to filter them for the sleep examination [3].

Although muscle artifacts are faster than EEG signals, there is some overlap in the frequency domain, particularly with pathological signals. Therefore, conventional digital filters cannot be used to remove artifacts without distorting the cerebral activity. An attractive solution is spatial filtering based on regression methods (for review see [4]), principal component analysis (PCA) [5], independent component analysis (ICA) [6–9], or canonical correlation analysis (CCA) [10, 11].

In this paper, dual adaptive filtering by optimal projection (DAFOP) is proposed to filter muscle artifacts. The DAFOP method was introduced in our previous work [1] to filter electrode artifacts on EEG recordings. The adaptation to the filtering of muscular artifacts requires a specific development in order to optimize the method for preserving cerebral signals, particularly those characterizing epilepsy. DAFOP method is a frequency dual application of the standard AFOP method, previously introduced by our team [12] which had also been used to filter muscle artifacts. DAFOP is designed to improve the results of AFOP by better preserving EEG while always highly reducing EMG artifacts. In addition, DAFOP brings the following advantages.

- Subjects do not have to perform the prerecording of two minutes at the beginning of each session to detect the spatial localization of the artifacts.
- The level of filtering adapt as a function of the number and amplitude of artifacts. Thus, DAFOP does not remove signals when there is no artifact.
- DAFOP is not limited on the number of possible artifact sources, contrary to AFOP which filters only the artifact sources experienced during the training period.

DAFOP combines spatial and frequency filtering. The principle consists in comparing two frequency windows with common spatial pattern (CSP) in order to identify brain sources using an a priori defined frequency power distribution. The entire EEG is then independently rebuilt by applying a regression method to various frequency windows. Because the optimal choice of frequency windows is problematic, a semiautomatic process is proposed to obtain the best settings. DAFOP is then evaluated through visual analysis of clinical EEGs and compared to two other methods: BSS-CCA [11] and a standard low-pass filter.

## 2. Methods

### 2.1. The DAFOP Method

Let **X** (dimensions (*n*, *T*)) be the signal matrix where the *n* rows represent the channels and the *T* columns represent the time samples. The aim of DAFOP is to construct a spatial filter which can be represented by a filtering matrix **F**  (*n*, *n*). The filtered signal **X**′  (*n*, *T*) will be given by application of the filtering matrix (**X**′ = **F** **X**).

As in all methods of spatial filtering, **F** is defined to conserve as much as possible cerebral sources while eliminating artifact sources. Before detailing the definition of **F**, a process of frequency window decomposition is presented to expand the possibilities of source separation.

#### 2.1.1. Frequency Window Decomposition

Artifact and cerebral signals are not always activated together and some may only belong to a specific frequency window. Since, in practice, the number of artifact and cerebral sources is far superior to the number of channels, attempting to filter an artifact which is not actually present leads to a small diminution in cerebral signal and trying to maintain a weak cerebral signal leads to the maintaining of a small portion of artifacts. In previous works [1, 12], we proposed applying a spatial filter adapted to individual time-frequency windows decomposing the signal. Thus, the spatial filters are only optimized for sources within the concerned frequencies. A frequency window Φ of multichannel signals corresponds to each channel extracted from a period of time and filtered within a frequency window Φ.

Signal decomposition consists in working within a temporal sliding window (corresponding to the matrix **X** discussed below) and a set of disjoint frequency windows *Ω* = {Φ} so that the sum of all frequency windows corresponds to the original signal:

(1) $$\begin{array}{c} \underset{\Phi \in \Omega }{\sum }X^{\Phi }=X \end{array}$$

with **X** ^Φ^ corresponding to the extraction of the frequency window Φ on **X**. Once this decomposition is defined, a different spatial filter can be applied to each window. Each of those spatial filters will be the result of a DAFOP process, with specific optimization for the frequency window. The artifact-free signals will then correspond to the sum of all windows which are spatially filtered:

(2) $$\begin{array}{c} X^{\prime }=\underset{\Phi \in \Omega }{\sum }F^{\Phi }X^{\Phi } \end{array}$$

with **F** ^Φ^ being the specific filtering matrix with a specific rank *n*_1_^Φ^ for the time-frequency windows **X** ^Φ^.

The construction of **F** ^Φ^ is then divided into two steps:

- construction of **W**_1_^Φ^  (*n*_1_^Φ^, *n*) a separation matrix of cerebral sources,
- construction of **M**_1_^Φ^  (*n*, *n*_1_^Φ^) a mixing matrix of cerebral sources.

**F** ^Φ^ is then defined by

(3) $$\begin{array}{c} F^{\Phi }=M_{1}^{\Phi }W_{1}^{\Phi }, \end{array}$$

where **W**_1_^Φ^ is defined by optimization of a frequency pattern through common spatial pattern (CSP), whereas **M**_1_^Φ^ is defined by linear regression on a sliding frequency window.

#### 2.1.2. Step 1: Estimation of **W**_1_^Φ^

This first step is common to all frequency windows inside a temporal sliding window **X**. It is assumed that, inside this time window, the spatial distribution of artifacts is the same for all frequencies. In practice, this may not be perfectly true but the effect seems negligible compared to other hypotheses. Considering the location of artifacts as constant is equivalent to considering the separation of cerebral sources as constant [1]. The aim of this first step is to estimate this separation by an optimization of a specific frequency pattern.

A frequency pattern is defined by two frequency windows Φ_cer_ and Φ_art_ which are chosen so that the power of a cerebral source is maximal in Φ_cer_ and minimum in Φ_art_, whereas the power of an artifact source is minimal in Φ_cer_ and maximum in Φ_art_. For a separation row vector **w**, we define the variance ratio *ρ*(**w**) as

(4) $$\begin{array}{c} \rho \left(w\right)=\frac{{||wX^{\Phi_{\text{cer}}}||}^{2}}{{||wX^{\Phi_{\text{art}}}||}^{2}}. \end{array}$$

The cerebral component separation **W**_1_^Φ^ is chosen to correspond to the vectorial subset maximizing this ratio. This problem can be solved using the CSP method [1] by defining **W**_1_^Φ^ as the *n*_1_^Φ^ eigen vectors with greatest eigen values of (**X** ^Φ_art_^ **X** ^Φ_art_^ ^*T*^)^−1^ **X** ^Φ_cer_^ **X** ^Φ_cer_^ ^*T*^.

#### 2.1.3. Step 2: Estimation of Cerebral Source Distribution **M**_1_^Φ^

The second step of DAFOP consists in determining the mixing subspace of cerebral sources using a linear regression method on **X** ^Φ^. Let us note **C** ^Φ^ = **X** ^Φ^ **X** ^Φ^ ^*T*^/*T* being the covariance matrix.

The aim of the regression is to find the mixing matrix **M**_1_^Φ^ which minimizes the squared difference between the filtered window and the original raw data windows:

(5) $$\begin{array}{c} M_{1}^{\Phi }=\underset{M}{\arg\;\min}\underset{i}{\sum }{||X_{i}^{\Phi }-MW_{1}^{\Phi }X_{i}^{\Phi }||}^{2} \end{array}$$

with **X**_*i*_^Φ^ corresponding to the *i*th time sample of **X** ^Φ^. This is a standard linear least squares problem and the solution is obtained by [12]

(6) $$\begin{array}{c} M_{1}^{\Phi }=C^{\Phi }{W_{1}^{\Phi }}^{T}{\left(W_{1}^{\Phi }C^{\Phi }{W_{1}^{\Phi }}^{T}\right)}^{-1}. \end{array}$$

The corresponding filtering matrix is thus

(7) $$\begin{array}{c} F^{\Phi }=M_{1}^{\Phi }W_{1}^{\Phi }=C^{\Phi }{W_{1}^{\Phi }}^{T}{\left(W_{1}^{\Phi }C^{\Phi }{W_{1}^{\Phi }}^{T}\right)}^{-1}W_{1}^{\Phi }. \end{array}$$

### 2.2. BSS-CCA and Equivalence with DAFOP

BSS-CCA is another method to filter muscular artifacts [2, 11, 13] and it seems to be one of the most efficient methods. Then, it is interesting to compare the results of this method with those of the proposed approach. In addition, except for a few details, this method can be considered as a particular case of DAFOP.

The BSS-CCA algorithm aims to find the most autocorrelated sources. For a discrete signal *s*(*kt*) (*t* being the sampling period and *k* = 1,…, *K* the sample number), the autocorrelation is defined as *p*_*s*_ = ∑_*k*_ *s*(*kt*)*s*((*k* − 1)*t*). For a set of *n* discrete signals (which corresponds to the *n* EEG channels) *x*_*i*_ (*i* = 1,…, *n*), it is noted that **X**_*k*_ = (*x*_1_(*kT*),…, *x*_*n*_(*kT*))^*T*^ the *k*th time sample of the multi-channel signals **X**. The problem of BSS-CCA consists then in determining **w** = arg max⁡_**w**,||**w**||=1_∑_*k*_ **w** ^*T*^ **X**_*k*_ **X**_*k*−1_^*T*^ **w**. According to [2], this is nearly equivalent to the problem **w** = arg max⁡_**w**_1_,||**w**_1_||=1_max⁡_**w**_2_,||**w**_2_||=1_∑_*k*_(**w**_1_^*T*^ **X**_*k*_ **X**_*k*−1_^*T*^ **w**_2_). This last problem can be solved by CCA thanks to the eigenvalue decomposition of

(8) $$\begin{array}{c} \Sigma_{X_{k}X_{k}}^{-1}\Sigma_{X_{k}X_{k-1}}\Sigma_{X_{k-1}X_{k-1}}^{-1}\Sigma_{X_{k-1}X_{k}}=PDP^{-1} \end{array}$$

with Σ_**X**_*k*_**Y**_*k*__ = ∑_*k*_ **X**_*k*_ **Y**_*k*_^*T*^/*K* corresponding to the cross-covariance matrix, **D** being the diagonal matrix of eigen values sorted by decreasing order, and **P** being the matrix of eigen vectors. The first vectors of **P** will correspond to the less autocorrelated sources and the last ones will correspond to the most autocorrelated ones. De Clercq et al. have observed that muscular artifacts correspond to these less autocorrelated components and the cerebral signal corresponds to these most autocorrelated components (“[*⋯*] brain activity produces structured signals having a high autocorrelation, whereas muscle activity is less structured and encompasses more properties related to temporally white noise.” [10]).

**Figure 2:**
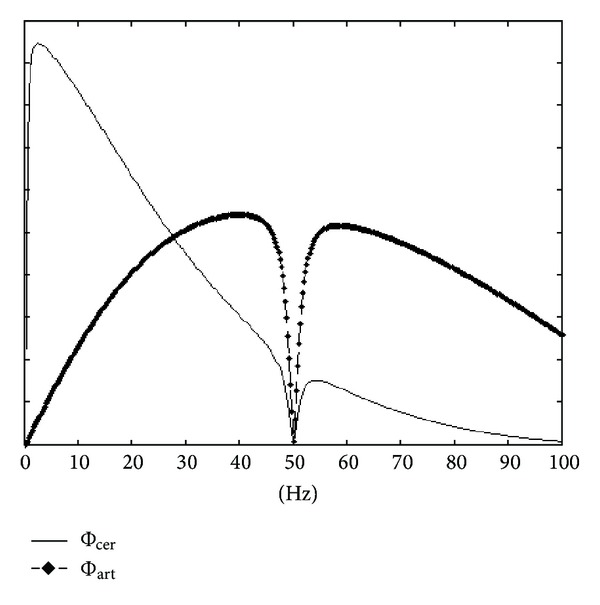
The two frequency windows used for DAFOP optimization in order to have CCA equivalence. The windows take into account the preprocessing of the recording proposed in [2] (i.e., high pass at 0.3 Hz, low pass at 35 Hz, and notch filter at 50 Hz).

If we neglect border effects by considering **X**_0_ = **X**_*K*_, the following three statements are equivalent:

1. **w** = arg max⁡_**w**,||**w**||=1_∑_*k*_ **w** ^*T*^ **X**_*k*_ **X**_*k*−1_^*T*^ **w**;
2. **w** is the eigen vector corresponding to the higher eigen value of (Σ_**X**_*k*_**X**_*k*__^−1^Σ_**X**_*k*_**X**_*k*−1__^sym^)^2^, with Σ_**X**_*k*_**X**_*k*−1__^sym^ = (1/2)(Σ_**X**_*k*_**X**_*k*−1__ + Σ_**X**_*k*−1_**X**_*k*__) (in practice, there is almost no difference with (8) since the matrix Σ_**X**_*k*_**X**_*k*−1__ is nearly symmetrical);
3. **w** is the result of DAFOP with windows Φ_cer_ and Φ_art_ corresponding to Fourier filters with $\Phi_{\text{cer}}(f)=\sqrt{1-\cos(2\pi fT)}$, Φ_art_(*f*) = 1 − Φ_cer_(*f*) and without frequency decomposition (i.e. **X** ^Φ^ = **X**).  Figure 2 illustrates these two filters.

The equivalence between (1) and (3) comes from the fact that, for any discrete signal *x*_*k*_  (*k* = 0,…, *K*) of sampling period *T* and periodic of period *KT*,

(9) $$\begin{array}{c} \overset{K}{\underset{k=1}{\sum }}x_{k-1}x_{k}=\overset{K}{\underset{k=1}{\sum }}x_{k}x_{k}-\overset{K}{\underset{k=1}{\sum }}{\left(x*\phi \right)}_{k}^{2}, \end{array}$$

where *ϕ* is the inverse Fourier transform of $\Phi (f)=\sqrt{\left(1-\cos(2\pi f/T\right)}$ sampled at period *T* and ∗ corresponds to the convolutional product. The equivalence is obtained by setting *x*_*k*_ = **w** ^*T*^ **X**_*k*_.

The BSS-CCA method is an efficient method to filter muscle artifacts. However, it would be possible to use the frequency decomposition in order to remove more components from artifacted frequencies and to keep more components in nonartifacted frequencies. In addition, it would be reasonable to hypothesize that setting Φ_cer_ and Φ_art_ could be optimized. This is the subject of the next section.

### 2.3. DAFOP to Filter Muscle Artifacts

In order to apply DAFOP to muscle artifacts, it was necessary to define a set of parameters, namely:

- frequency window decomposition *Ω* = {Φ};
- the two frequency windows Φ_cer_ and Φ_art_;
- the number of components to conserve within each window *n*_1_^Φ^.

**Figure 3:**
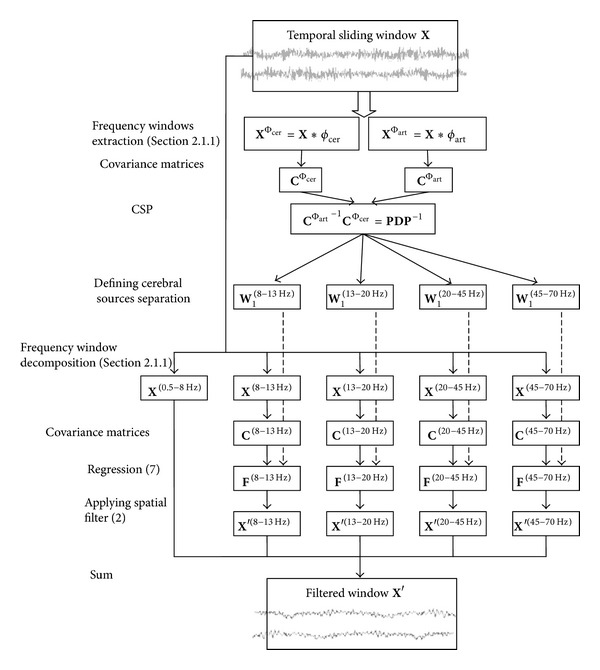
Steps of the DAFOP method to filter muscle artifacts.

Figure 3 sums up the global method of artifact filtering and the various parameters which influence filtering.

To set these parameters, a training dataset of 12 clinical EEG recordings of different patients was selected. Recordings were performed at the Hospital Group GHICL (Groupe Hospitalier de l'Institut Catholique de Lille) and the Hospital Center of Lille, France, using Nihon Kodhen, Nicolet, and Micromed devices. The electrodes were positioned according to the 10/20 system with 19 electrodes. Preprocessing consisted of a common mean reference, a high-pass filter at 0.5 Hz, a low-pass filter at 70 Hz, and a notch filter at 48–52 Hz (power line frequency). These filters were 6-order Butterworth (12 for the notch) applied with a forward-backward process to prevent phase shifting. Recordings included, among other signals, some epilepsy seizures, and pathological rhythms (spikes, spike waves, etc.). Several trials were run on this dataset to adjust the various parameters. The setting was done either subjectively or by optimization, in order to best remove muscle artifacts without erasing cerebral rhythms. The next paragraphs explain these choices.

#### 2.3.1. The Frequency Decomposition

Frequency decomposition *Ω* = {Φ} was empirically established to the following windows: 0–8 Hz, 8–13 Hz, 13–20 Hz, 20–40 Hz, and 40–70 Hz. The ratio of cerebral rhythms/muscular artifacts within each of these frequency windows was almost constant but different between windows.

No muscle artifacts are present in the frequency band 0–8 Hz. Consequently, it is not necessary to remove components and the signals can remain unchanged. For the other bands, the artifact ratio increased with frequency (Figure 1). Thus, it is preferable to increase the number of removed components on higher frequency bands (see Section 2.3.3).

#### 2.3.2. Choice of Windows Φ_cer_ and Φ_art_

*(a) General Idea*. The first step of DAFOP determines the spatial distribution of artifact sources by comparing two frequency windows. These two frequency windows Φ_cer_ and Φ_art_ are represented by convolutional filters of indicial response *ϕ*_cer_(*t*) and *ϕ*_art_(*t*). These two filters must be defined so that **X** ^Φ_cer_^ carries more cerebral signal whereas **X** ^Φ_art_^ carries more artifacts.

As shown in Figure 1, muscle artifacts account for a major portion of power at high frequencies, with the power decreasing down until 8 Hz. For cerebral sources, this depends on the signal. The constants correspond to a major persistent part of the power on the alpha band and the lower frequencies and a minor (or null) part of the power at high frequencies.

The signals within the frequency band 0 to 8 Hz are not changed at all since there are no muscle artifacts in this band. Consequently, cerebral sources carrying theta and delta waves are not needed.

Taking into account these observations, an initial empirical choice could be **X** ^Φ_cer_^ corresponding to the band (8–13 Hz) (alpha) and **X** ^Φ_art_^ corresponding to the band [30–70 Hz]. However, our trials indicate that this choice would still be suboptimal. Thus, we propose a method to find an optimization of windows Φ_cer_ and Φ_art_.

*(b) Construction of Training Signals from Collected Data*. Two monochannel signals *s*_cer_ and *s*_art_ were constructed. These signals were extracted for certain periods and certain channels from a training dataset of recordings.

*s*_cer_ was made out to be the cleanest possible cerebral signal. It corresponded to a concatenation of various rhythms covering as much as possible the variety of possible waves in the frequency bands >8 Hz. It is composed of the following rhythms: *α*, *μ*, *β*, fast ripples, spikes, spike waves, polyspikes, and vertex waves.

*s*_art_ was made out to be the cleanest possible muscle artifact. It corresponded to a concatenation of muscle artifacts on channels and periods where cerebral signals were negligible compared to artifact signals.

**Figure 4:**
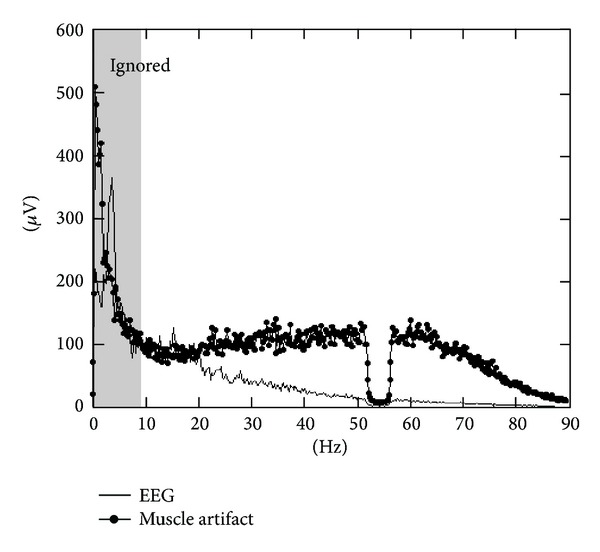
Smoothed Fourier transform module for the two training signals.

The selected periods last about 20 s by signal. In order to represent the wide variety of possible cerebral signals and muscular artifacts, approximately 100 periods were included in the selection for each signal. At the end, two signals of approximately 30 min duration each were obtained.  Figure 4 shows the Fourier transform module and confirms that there is not a clear frequency limit which separates cerebral signals from muscle artifacts. Consequently, any frequency filter could eliminate all artifacts while preserving all cerebral signal.

The two signals are then preprocessed using a high-pass filter with a cutoff at 8 Hz so signals (artifact and cerebral) below these bands would not interfere with the filter settings. Indeed, most muscle artifacts are associated with electrode artifacts since the muscle contraction drives a facial movement. Nevertheless, the electrode artifact distribution is not directly linked to the electromyographic artifact distribution. It is therefore important to ignore the electrode artifact when determining muscle artifact distributions.

*(c) Convolutive Filter Optimization*. Using the two previously defined signals *s*_cer_ and *s*_art_, we look for an optimization of the frequency windows Φ_cer_ and Φ_art_. We note the inverse Fourier transform *ϕ*_cer_ and *ϕ*_art_, corresponding to convolutional filters. Optimal filters are then defined so that the power of *s*_cer_ convolved by the filter *ϕ*_cer_ is maximum whereas the power of *s*_cer_ convolved by *ϕ*_art_ is minimum. Inversely, the power of *s*_art_ convolved by *ϕ*_cer_ is minimum whereas the power of *s*_art_ convolved by *ϕ*_art_ is maximum:

(10) $$\begin{array}{c} \phi_{\text{cer}}=\underset{\phi }{\arg\;\max}\frac{||\phi *s_{\text{cer}}||}{||\phi *s_{\text{art}}||}\\ \phi_{\text{art}}=\underset{\phi }{\arg\;\min}\frac{||\phi *s_{\text{cer}}||}{||\phi *s_{\text{art}}||} \end{array}$$

which can also be written as

(11) $$\begin{array}{c} {\vec{\phi }}_{\text{cer}}=\underset{\vec{\phi }}{\arg\;\max}\frac{||{\vec{\phi }}^{T}S_{\text{cer}}||}{||{\vec{\phi }}^{T}S_{\text{art}}||}\\ {\vec{\phi }}_{\text{art}}=\underset{\vec{\phi }}{\arg\;\min}\frac{||{\vec{\phi }}^{T}S_{\text{cer}}||}{||{\vec{\phi }}^{T}S_{\text{art}}||} \end{array}$$

with $\vec{\phi }$ designating the column vector formed by the time samples of *ϕ* and **S**_*i*_ designating the signal matrix defined by **S**_*i*__*j*,*k*_ = *s*_*i*_((*k* + *j* − 1)*T*_*s*_) (*T*_*s*_ being sampling period).

*(d) Resolution*. The common spatial pattern method which consists of computing the two covariance matrices **C**_**S**_cer__ = **S**_cer_ **S**_cer_^*T*^ and **C**_**S**_art__ = **S**_art_ **S**_art_^*T*^ was used. Thereby, ${\vec{\phi }}_{\text{cer}}$ was defined as the eigen vector of the highest eigen value of **C**_**S**_art__^−1^ **C**_**S**_cer__ and ${\vec{\phi }}_{\text{art}}$ as the eigen vector of the lowest eigen value.

Due to frequency preprocessing of *s*_cer_ and *s*_art_, the rankings of **C**_**S**_cer__ and **C**_**S**_art__ are not complete (many eigen values are close null). This can lead to *ϕ*_cer_ and *ϕ*_art_ corresponding to frequencies with almost no signal which will be irrelevant and unstable. Consequently, a principal component analysis (PCA) was performed prior to CSP to reduce the research of components only on relevant frequencies. This corresponds to the principle of dimension reduction generally used in blind source separation [14].

**Figure 5:**
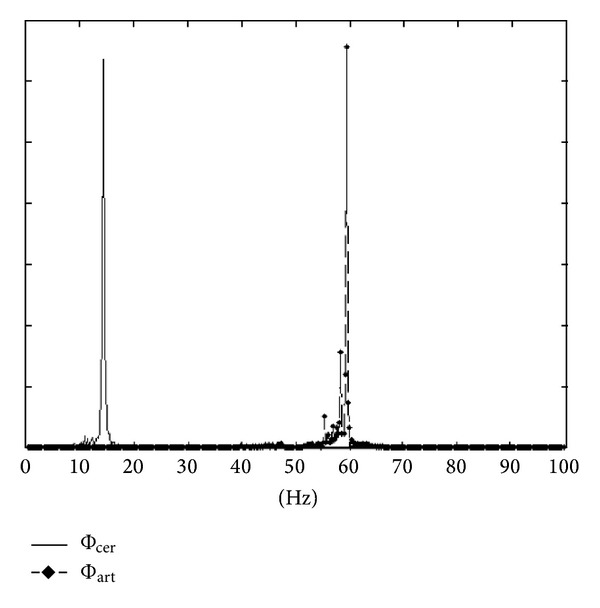
Frequency windows of DAFOP filtering obtained by FIR optimization.

*(e) Resulting Filters*.  Figure 5 illustrates the Fourier transform of the two windows through this optimization. The two windows are almost Dirac; Φ_cer_ at 13 Hz and Φ_art_ at 60 Hz. After various trials on the training dataset, it seemed that this setting was indeed the best to separate muscle artifact sources from cerebral sources.

#### 2.3.3. Number of Conserved Components

The last parameter to set is the number of conserved components *n*_1_^Φ^ as a function of the time frequency windows **X** ^Φ^. This number can be set to the number of components of variance ratio *ρ* (4) greater than a threshold *t* ^Φ^. If there is no artifact in a current period, the ratio of all components would be high and no components would be removed. If there are many artifacts in the current period, the ratio of some components would be low and several components would be removed.

Furthermore, since there are more artifacts at high frequencies, the number of removed components should increase with frequency. This is why the threshold *t* ^Φ^ will be higher for high frequency windows. In contrast, concerning the frequency window 0.5–8 Hz, there are no muscle artifacts and no components are removed. The threshold *t* ^0.5–8 Hz^ can then be set to 0.

Ideally, a component should be removed when it contains more than a certain percentage of artifacts (around 70% for our objective). Unfortunately, the artifact ratio is unknown but it could be estimated in function of *ρ* and the frequency window Φ. Consequently, we have set empirically *t* by observing components on the various frequency windows associated with their *ρ* ratio. This parameter is not very sensitive and important threshold variation is necessary to observe significant differences. A greater value would imply removing more artifacts but removing also more cerebral signals. In new studies, we expect to define various sets of parameters in order to allow EEG readers to adapt the level of filtering on the application.

### 2.4. Method for Evaluation on Clinical Recordings

An evaluation by an expert neurologist was conducted in order to compare the results with other methods described in the literature and to evaluate the results in using this method in routine clinical practice.

#### 2.4.1. Data Collection

Clinical recordings of 20 epileptic patients with pertinent cerebral rhythms were selected. These recordings were different from those of the training dataset but were acquired under the same conditions. They lasted from 20 minutes (short duration recordings) to 4 days (long duration recordings). Firstly, 114 relevant pages were selected among the 20 recordings without viewing the filtered result. One page corresponded to a 20 s epoch of EEG with the 19 channels according to the 10/20 system. The selection was done with respect to various levels of artifact power and for a wide variety of cerebral signals. Coauthor neurologists selected EEG pages from their own patients. Selected pages were anonymized. As such, no specific assessment was necessary.

The three following filters were then compared:

- a standard 1-order low-pass filter at 30 Hz common to many EEG device software applications,
- a filter achieved with BSS-CCA [10, 13],
- a DAFOP filter with the above optimized parameters.

For this study, we used a previously published program (http://www.neurology-kuleuven.be/?id=210) distributed by the authors of BSS-CCA. Concerning the number of components to remove or to conserve, De Clercq et al. proposed a manual selection by specialists [10, 11]. However, we found important to compare only entirely automated methods. De Clercq et al. have suggested that thresholding the autocorrelation index might be sufficient to remove muscle artifacts automatically within a large subject group. After several trials on our training dataset of 12 recordings, we found that setting a square autocorrelation threshold at 0.88 gives almost the same results as the expert selection.

#### 2.4.2. Evaluation Methods

Two expert neurologists compared the filtered signals of these EEG pages. For each page, the filtering results of the three methods were presented at random, so experts performed a blinded analysis, thus reducing subjectivity.

Each expert analyzed one half of the dataset. For each page, the raw recording was first interpreted. The experts evaluated the presence of cerebral activities with the following categories (spikes, spike waves, alpha, pathological rhythmic discharges, spindles, and vertex sharp waves). In addition, they examined the original amount of muscle artifacts and scored it from 0 to 4 (0 = no artifact; 4 = very high level of artifacts).

Thereafter, the experts analyzed the results of each filtering method. They scored the proportion of removed muscle artifacts from 0 to 4 for (0 = no amplitude reduction (<10%); 1 = 10–35%; 2 = 35–65%; 3 = 65–90%; 4 = complete elimination (>90%)) and the proportion of reduced cerebral activities from 0 to 4 (0 = unchanged activities (<10%); 1 = 10–35%; 2 = 35–65%; 3 = 65–90%; 4 = no identifiable activities (>90%)). The average ratio estimation is calculated by considering the middle of the bins of each score:

(12) $$\begin{array}{c} \text{Avg}=\frac{5n_{0}+22.5n_{1}+50n_{2}+77.5n_{3}+95n_{4}}{100\left(n_{0}+n_{1}+n_{2}+n_{3}+n_{4}\right)} \end{array}$$

with *n*_*i*_ being the number of signals on which the experts have assigned a mark *i*. Concerning electromyographic artifact elimination, it is more important to filter artifacts when they are at a high level since they prevent interpretation. Consequently, the electromyographic artifact elimination estimator is weighted by the level of artifacts given by the expert on the raw recording, and thus a closer estimation of the global amount of removed artifacts was obtained.

Finally, experts have balanced all criteria to determine for each page the most efficient method. Balancing takes into account the muscle artifact elimination, the proportion of reduced cerebral activities, and the artifact addition or modification. Since the signal belonging to the frequency band 0–8 Hz was not modified, the slow waves were not reduced. Consequently, the reduction was not subject to evaluation. However, experts could consider a given page to be better if the slow waves were more visible after filtering.

Statistical sign tests were done to compare CCA and DAFOP for each parameter and for all balanced criteria comparison. The *P* value for significance was fixed at 5%.

## 3. Results

**Figure 6:**
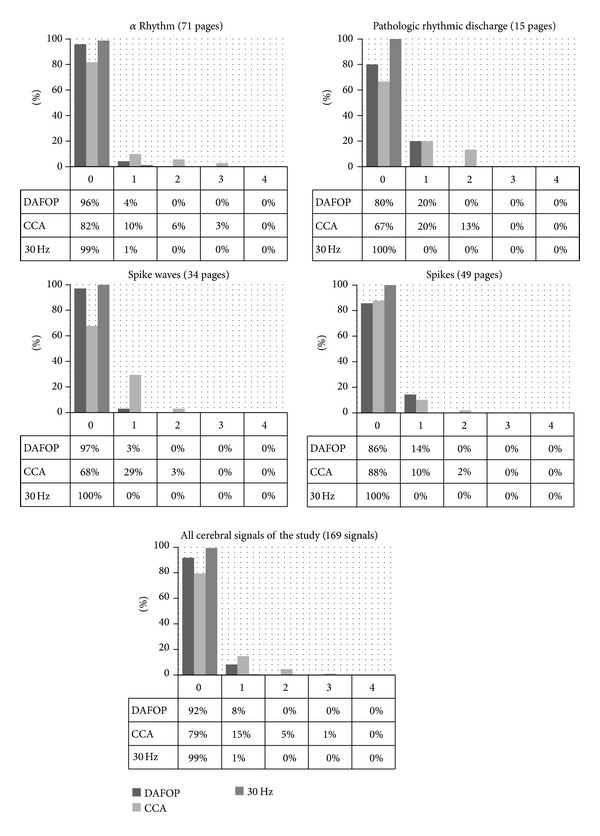
Amount of removed cerebral signals per 20 s page (0: no difference (<10%); 1: (10–35%); 2: (35–65%); 3: (>65–90%); 4: no longer identifiable (>90%). For example, the table can be read as follows: among the 71 pages with an alpha rhythm, the experts noticed no significant difference in the alpha signals on 96% of pages.

Table 1 displays the estimation of average ratios of artifact/cerebral signal elimination and Figure 6 displays the distribution of the scores given by the two experts for each type of cerebral signals.

Regarding preservation of cerebral activity, the 30 Hz filter had the best performance (elimination of 5.1% of cerebral activity), followed by DAFOP (6.45%) and lastly by CCA (10.58%). For CCA, these differences were pronounced for alpha rhythms, epileptic rhythmic discharges, and spike waves. Figure 6 shows that the signals were never completely removed by any of the methods. There were only 5% and 1% of signals showing, respectively, a moderate (score 2) and an important reduction (score 3) of cerebral activities for the CCA method and none for the others. For the other signals, with DAFOP and CCA, the reduction score was 0 for the majority and 1 for a small proportion.

Concerning elimination of electromyographic artifacts, DAFOP performed the best (84.29% elimination), then CCA (82.28%), and lastly the 30 Hz filter (55.51%) which is clearly less efficient than the first two methods. However, in the four cases analyzed with DAFOP and the case analyzed with CCA (none for 30 Hz filter), an added or transformed artifact could have been interpreted as a pathological signal, if the filtered EEG was analyzed alone. Moreover, on 10% of pages for DAFOP and 3% for CCA, the muscle artifact residue could be confounded with alpha rhythm.

As on the original assumption, the experts did not notice any significant differences on the delta and theta rhythms, in the 23 concerned pages.

**Figure 7:**
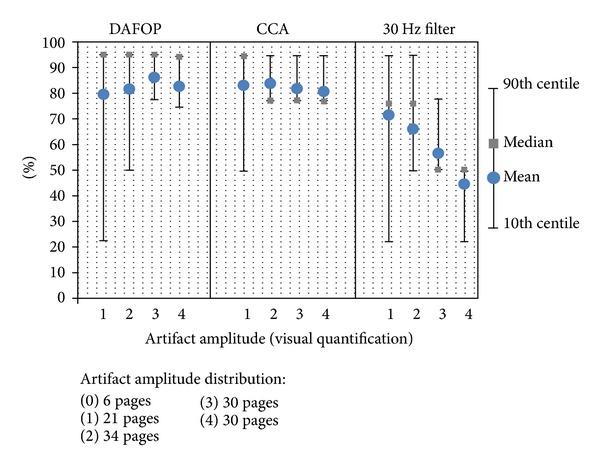
Distribution of the estimated amount of removed artifacts versus artifact amplitude for each method.

Figure 7 presents the amount of removed artifact as a function of artifact amplitude. The 30 Hz filter worked least well (43% of elimination of artifacts at level 4) when an important artifact was present, whereas DAFOP and CCA filters performed constantly higher (83% and 80%) even in the presence of an important amount of artifacts (level 4). DAFOP and CCA in few cases (3 pages for DAFOP and 1 page for CCA) were unable to efficiently filter (elimination 1 or 2) when artifact level was low (level 1). The mean ratio of artifact removal was 80% for these cases which strongly suggests that these types of cases are less common.

Table 2 shows the results of the statistical comparison between DAFOP and CCA. A sign test was applied in order to determine the significance of the differences between the methods.

The statistical analysis shows that DAFOP was globally more efficient concerning both electromyographic artifact (*P* ≤ 0.026) elimination and conservation of cerebral signals (*P* ≤ 0.00019). According to blind expert analysis and taking into account all parameters, DAFOP was globally more efficient than CCA (*P* ≤ 0.00575) in our tests.

**Figure 8:**
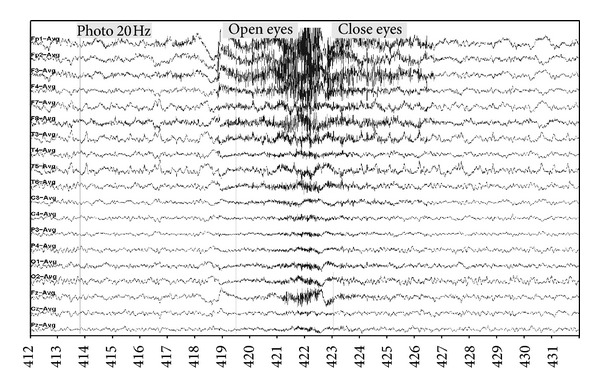
Example of Raw EEG signal with important muscle artifact (level 3/4), including α rhythm and spikes (F7, T3, T5).

**Figure 9:**
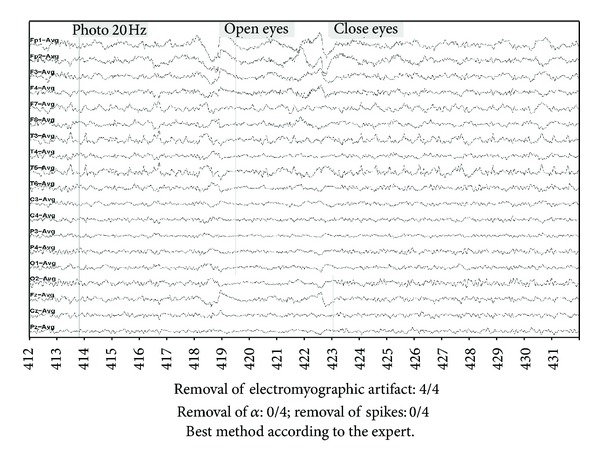
Filtering result with DAFOP method.

**Figure 10:**
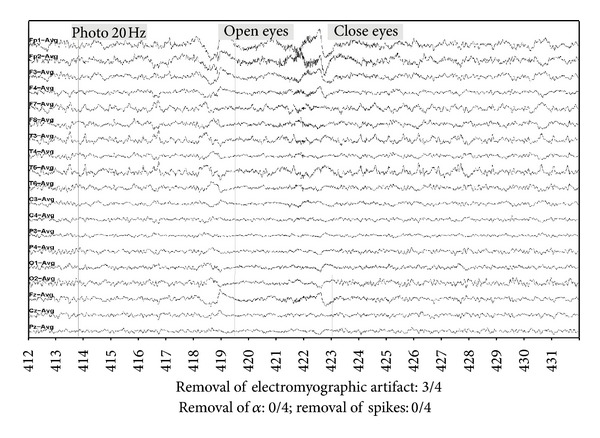
Filtering result with CCA method.

**Figure 11:**
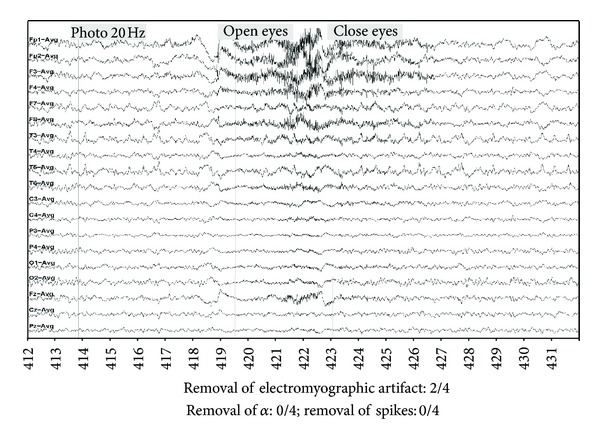
Filtering result with a 30 Hz filtering.

An example of the results obtained with the three methods is given in Figures 8, 9, 10 and 11 as well as the corresponding neurologist evaluation. An important muscle artifact can be observed on the frontal area between 418 and 427 seconds (Figure 8). This artifact was judged as completely eliminated (score = 4) with the DAFOP method (Figure 9), highly reduced (score = 3) with the CCA method since there remains a small muscular activity on Fp1 and Fp2 (Figure 10), and moderately reduced (score = 2) with the 30 Hz filter (Figure 11). No reduction was observed with any of the methods for both spikes (channels T3, T5, and F7) and alpha rhythm. The expert judged that DAFOP allowed to get the best result in this example. It can further be noticed that there were ocular artifacts on seconds 418 and 422 but these artifacts are outside the scope of this evaluation.

## 4. Discussion

### 4.1. Performance Comparison between Filtering Methods

DAFOP and CCA both gave promising results for the elimination of electromyographic artifacts on EEG recordings and both offer a high selectivity concerning the conservation of normal and pathological cerebral signals. On average, the filtering rate was 84.3% and 82.3%, respectively, for muscle artifacts whereas for cerebral signals it was 5.7% for DAFOP and 11.3% for CCA (Table 1). In addition, both methods never completely removed the cerebral signals (Figure 6). Only 3% of the alpha rhythms showed important reductions (>65%) with CCA and none with DAFOP.

For the three methods, the reduction scored by neurologist always corresponded to an amplitude reduction of signals and never to a signal deformation. However, it can be noticed, however, that for the 30 Hz filter and the DAFOP filter the high frequencies can be a bit more reduced than the low frequencies. Consequently a spike wave will have his spike slightly more reduced than the wave. Nevertheless, this effect was very low and it would be difficult to quantify it visually.

DAFOP transformed, in some less frequent cases, artifacts into signals which could be misinterpreted as cerebral signals by inexperienced readers. Artifact transformation also arises with CCA but to a lesser extent. Although the DAFOP filter could be placed at a stronger setting to remove those signal addition/transformation, our priority was to optimize conservation of cerebral signals. Taking into account that both unfiltered and filtered signals are analyzed by physicians, signal addition does not represent a real life limitation and as such the proposed settings are optimized with these results in mind.

Regarding electromyographic elimination, the efficiency of both DAFOP and CCA is almost independent of the amount of artifacts (Figure 7). However, in some rare cases where artifacts are low, they may rest almost unfiltered. In any case, in these situations interpretation is not hindered by artifacts. This situation raises more concern with the DAFOP method but can also occur with CCA.

The 30 Hz low-pass filter is a conventional filter used in clinical practice but is inefficient in the presence of important artifacts (Figures 7 and 11) but none the less conserves the cerebral signals. Theoretically, there is always a slight reduction, but not enough to change the expert's visual evaluation. Despite the advantages of this method, the experts judged that the 30 Hz filter rarely gave the best results (only three pages out of 114 examined, the three pages harboring few artifacts.)

Statistical comparison between DAFOP and CCA demonstrates that DAFOP method is better in conserving cerebral rhythms, particularly alpha rhythms and spike waves. DAFOP has also better achievement in electromyogram artifact elimination. Consequently even if the threshold of CCA would be changed, DAFOP will have a better selectivity. Finally, the general comparison of methods proves that even if there can be artifact addition/modification, DAFOP overall gives better results than CCA.

Concerning the method functioning, DAFOP and CCA are both based on the separation of components which optimize frequency pattern and this seems to be an efficient criterion. However, the CCA frequency patterns are not directly optimized for the problem but result from methodological simplification which could explain partially the better results of DAFOP. The other explanation for the DAFOP superiority is frequency decomposition which increases the possibility of source separation. Using CCA with this frequency decomposition should give also good results.

### 4.2. Comparison with Other Methods Referenced in the Literature

It would be interesting to compare the DAFOP method with other methods referenced in the literature like standard AFOP [12], ICA [15], higher order, and wavelet filter [16]. However, we can already make an assessment on performance and limitations of these methods but ideally a blind comparison by third persons should be done to validate them.

In relation to standard AFOP, according to the parameters and the results in previous studies [1] and to our trials, AFOP would have approximately the same level of artifact removing from DAFOP (86% for AFOP against 84% for DAFOP). Nevertheless, AFOP removes more cerebral signals (the average reduction of spikes was judged 18.8% for AFOP against 7.5% with DAFOP with similar evaluation methods). However, these observations have to be confirmed by a blind analysis. The separation criterion of AFOP is based on spatial localization of component whereas DAFOP is based on a frequency pattern. Then, it could be envisaged to combine both methods to improve their performances.

In regard to ICA, we have previously done a comparison between standard AFOP and manual ICA [1] (Infomax method [17]). Standard AFOP was judged to better remove EMG artifacts in almost all cases (in 49 cases, only 2 pages were in favor of ICA while 40 were in favor of AFOP). ICA seemed to have more difficulties to separate EMG from cerebral signals. However, this study included the filtering of other types of artifacts which interfere with efficiency. There can also be other ICA methods like AMUSE [15] which may be more efficient than infomax to filter muscle artifacts.

Most of the time only first order filters are implemented in EEG reading devices and this is why we decided to compare with this. Higher order filter would probably be more efficient. However, due to the frequency distribution overlap between muscular artifacts and spikes (Figure 1) it would never be possible to eliminate artifacts while well preserving the interesting signal. Finally, some researchers work also on wavelet filters [16]. As far as we know, those filters have never been tested in presence of pathological signals and it can be supposed that the same kind of limitations may appear. However, wavelet can be combined with spatial filter with process similar to the frequency decomposition [18].

### 4.3. Evaluation Method

There are very few papers which demonstrate statistically that a filtering method is better than another [19]. Taking into account the wide variety of pathological signals and artifacts, it seems to us that this kind of evaluation is a necessity and too many papers focus only on a limited amount of pages.

The main potential bias of this study is that two experts do not represent the large variety of neurologist experts, and their opinions can be different from the reality. In addition, each of them only analyzed half of the database. However, since the significance is reached it will not change the conclusion and it will not remove the bias that there is only two experts. Nevertheless, it would be interesting to perform an interobservator comparison and verify the expert concordance. Such study has been realized in [20] including also ocular and electrode artifacts filtering.

Future studies should be done to complete these results by a qualitative and objective comparison on a synthetic signal, where the true cerebral signal is known. Some authors [3, 19] have proposed to select unartifacted periods and to add muscle artifact signals generated by the mixing of a few artifact sources. Those artifact source signals can be obtained by ICA on other periods or on other body muscles. Unfortunately, a realistic synthetic signal is difficult to construct and there can be two types of bias for the real performance estimation.

- It would not be possible to have a perfect unartifacted signal and a perfect muscle artifact source.
- Considering the artifact as a mixing of limited number of sources is not realistic for an important artifact. This model would ease the problem and particularly it would render the frequency decomposition process almost useless.

Despite these two problems, it would be interesting to verify that the conclusions are the same with such model.

Finally, this evaluation method mainly concerns visual examination of EEG, but it can be supposed that if the method is applied as preprocessing for other applications like source localization, anticipated detection of epilepsy seizures, brain computer interfaces, the optimal parameters, and the evaluation could be different.

### 4.4. Practical Aspects

From a practical point of view, all three methods are entirely automated. A turn of a switch moves from raw recording to filtered recording. It is not the case of methods like ICA [15] which requires manual selection of components or standard AFOP which requires that the patient carries out a specific protocol at the beginning of recording.

The methods can be applied to any EEG recording and do not require additional electrodes like regression methods [4].

In addition, all three methods have a very short computation time (<0.5 s for 20 s of signal on a dual core 2 GHz processor whereas ICA methods can take 10 s).

An advantage for DAFOP and to a lesser extent for CCA is that methods are stable if there is a disconnected or a missconnected electrode; that is, this electrode artifact will not be removed but the other channels will be still well filtered and the artifact will not be propagated on other channels. This is due to the fact that those kinds of signals are always uncorrelated to other channels on the concerned frequencies and the frequency pattern is very different of a muscular artifact. Sometimes, a power line artifact residue or another high frequency intrinsic instrument noise appears on an EEG channel. Even if the frequency pattern does not perfectly match, the power ratio *ρ* (4) is low enough to erase the corresponding component on the high frequency bands. The artifact is then removed.

Those advantages are particularly important on the context of standard EEG examination where recording have to be quickly analyzed.

## 5. Conclusion

This paper describes the use of the DAFOP method to filter muscle artifacts on EEG recordings and discusses optimization of the method. DAFOP was evaluated on clinical EEG recordings by two neurologists and compared with BSS-CCA and the 30 Hz low-pass filter. DAFOP was particularly efficient for artifact removal (84% on average) while offering very good conservation of cerebral signals (6.4% reduction on average), particularly pathological signals. Comparison with the 30 Hz filter commonly used in routine practice showed that the latter is far less efficient than DAFOP and BSS-CCA in enhancing EEG readability. In comparison with BSS-CCA, DAFOP was judged globally to be more efficient.

In addition to improving EEG readability, this method overcomes three drawbacks commonly reported in the literature [15].

- It does not require manual intervention.
- It has a low computational time enabling a neurologist to visualize for each second, 1 min of filtered EEG without previously processing the data.
- It works on any clinical EEG recording device without modifying current practice (no additional electrodes needed [21] for artifact recording and no additional protocol for individual patients [12]).

The method can also be combined with other methods to filter all types of artifacts. For example, DAFOP can be combined with AFOP [1] to filter electrode, ocular, and chewing artifacts and it can be combined with [22] to filter heartbeat artifacts. Due to their similarity in methodology, it is possible to combine them on more optimal way than the cascade filters, by simply adding the various covariance matrices. It can be noticed that muscle artifact filtering seems more efficient than the filtering of ocular and electrode artifacts with the similar methods [1].

This paper presents the using of DAFOP on the clinical context of EEG examination. Thus the method is parameterized and evaluated on this context. There would be many other applications of this filtering which probably require some small adjustments. For example, if the aim is to study the Fourier transform of EEG signal, the frequency decomposition step would add discontinuity on the gain multiplier. The Fourier transform of cerebral signal is already very discontinue and fortunately the discontinuity cannot be seen unless we observe the mean of several EEG spectra. The method should also be set and tested on other devices like magnetoencephalogram (MEG) and on EEG with more electrodes. Some adjustments should also be done for recording on countries with 60 Hz power line frequency. Finally, it would also be interesting to apply this method as preprocessing for other applications such as source localization, brain computer interfaces, and anticipated detection of epilepsy seizure [23].

For now, the possibility of implementing this method on clinical EEG devices is being studied.

## Figures and Tables

**Table 1:** Estimation of average ratios of artifact/cerebral signal elimination.

	DAFOP	CCA	30 Hz	Studied pages/signals
Estimation of the average ratio of cerebral signal elimination				
Alpha rhythm	5.74%	11.30%	5.25%	71
Epileptic rhythmic discharge	8.50%	14.50%	5.00%	15
Spike waves	5.51%	11.47%	5.00%	34
Spikes	7.50%	7.70%	5.00%	49
Spindles/vertex spikes	5.00%	5.00%	5.00%	6/3
Global	**6.45%**	**10.58%**	**5.10%**	**169**
Estimation of the average ratio of electromyographic artifact elimination	84.29%	82.28%	55.51%	108

**Table 2:** Blinded expert comparison of DAFOP and CCA filtering.

Comparison of	Sign test	Details	*P* value	Conclusion	Significance
Electromyogram elimination (all levels of artifacts)	Two-sided	DAFOP is better on 31 pages; CCA is better on 15 pages; same level on 67 pages.	*P* ≤ 0.0259	DAFOP has more often better electromyogriam elimination	Significant
Cerebral signal elimination (all cerebral signals of the study)	Two-sided	DAFOP is better on 30 signals, CCA is better on 7 signals; same level on 142 signals.	*P* ≤ 0.000191	DAFOP has more often better cerebral signal conservation	Highly significant
Alpha rhythm elimination	Two-sided	DAFOP is better on 12 signals, CCA is better on 1 signal; same level on 58 signals.	*P* ≤ 0.00342	DAFOP has more often better Alpha rhythm conservation	Highly significant
Spike elimination	Two-sided	DAFOP is better on 5 signals, CCA is better on 6 signals; same level on 38 signals.	*P* ≤ 1	DAFOP and CCA spike conservation seem similar	Not significant
Spike-wave elimination	Two-sided	DAFOP is better on 10 signals, CCA is better on 0 signal; same level on 24 signals.	*P* ≤ 0.00195	DAFOP has more often better spike-wave conservation	Highly significant
Epileptic rhythmic discharge elimination	Two-sided	DAFOP is better on 3 signals, CCA is better on 0 signals; same level on 12 signals.	*P* ≤ 0.25	DAFOP has better epileptic rhythmic discharge conservation	Not significant

Quality of filtering by pages, all criteria balanced	One sided	DAFOP is better on 58 pages; CCA is better on 33 pages; same quality on 23 pages.	*P* ≤ 0.00575	DAFOP is more often judged better than CCA when balancing all criteria	Highly Significant
